# Crystal Structures of a Cubic Tin(II) Germanate, α-Sn_6_GeO_8_, and a Tetragonal Tin(II) Silicate, γ-Sn_6_SiO_8_

**DOI:** 10.1021/acs.inorgchem.2c02053

**Published:** 2022-09-07

**Authors:** Daniel S. Parsons, Antony Nearchou, Joseph A. Hriljac

**Affiliations:** † Diamond Light Source, Harwell Science and Innovation Campus, Didcot, Oxfordshire OX11 0DE, U.K.; ‡ School of Chemistry, University of Birmingham, Edgbaston, Birmingham, West Midlands B15 2TT, U.K.

## Abstract

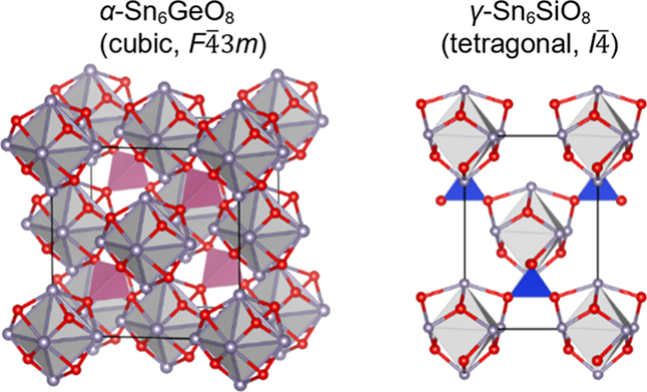

A cubic tin(II) germanate, α-Sn_6_GeO_8_ (space group *F*4̅3*m*, *a* = 10.52521(2) Å, and *Z* = 4), has been synthesized by both regular hydrothermal and microwave-assisted hydrothermal methods, and the crystal structure of this material has been solved by Rietveld refinement of synchrotron powder X-ray diffraction (PXRD) data. The crystal structure is analogous to α-Sn_6_SiO_8_ and is therefore related to the zinc blende structure comprising a face-centered cubic array of [Sn_6_O_8_]^4–^ anionic clusters with Ge^4+^ cations occupying half of the tetrahedral holes. Variable-temperature PXRD has revealed that tin(II) germanate has high thermal stability: remaining stable at 950 K and mostly decomposing over the range 984–1034 K. The tin(II) germanate has been further characterized by X-ray fluorescence (XRF), Raman, and diffuse reflectance (DR) UV–vis spectroscopies. In addition, variable-temperature PXRD studies have revealed the formation of a tetragonal tin(II) silicate polymorph, γ-Sn_6_SiO_8_ (space group *I*4̅, *a* = 7.30414(6) Å, *c* = 10.53731(6) Å, and *Z* = 2), at temperatures below 170 K. The crystal structure of γ-Sn_6_SiO_8_ has been elucidated by Rietveld refinement. While a transition to a tetragonal polymorph is observed upon cooling α-Sn_6_SiO_8_, no corresponding transition is observed for α-Sn_6_GeO_8_, which retains its cubic structure over the probed temperature range.

## Abstract 2

A tin(II) germanate, α-Sn_6_GeO_8_, has been synthesized, and the crystal structure of this material has been solved by Rietveld refinement of synchrotron powder X-ray diffraction (PXRD) data. The crystal structure of a tetragonal tin(II) silicate, γ-Sn_6_SiO_8_, has also been elucidated by Rietveld refinement.

## Introduction

In binary tin(II) oxides, such as the dominant form, blue-black SnO, and the metastable red SnO form, the structures comprise layers of edge- and corner-shared [SnO_4_] pyramids.^1,2^ In more complex solid systems containing tin(II), the divalent tin atoms often exhibit a propensity toward cluster formation, with tin(II)-containing clusters reported over a range of possible nuclearities and with significant variety in the potential bridging ligands.^3^ A frequently encountered tin(II) cluster contains an octahedral, or pseudo-octahedral, hexanuclear array of tin atoms with oxygen atoms bonded in a μ_3_-binding mode on each face of the octahedral array. These may be generally referred to as [Sn_6_O_8_] clusters, in which typically four of the oxygen atoms reside as discrete oxide ions in the cluster and the other four belong to a larger functionality. Several synthetic solid compounds contain discrete clusters of this type, Sn_6_O_4_(OR)_4_, in hydrogen-bonded arrays, where R = H,^4^ CH_3_,^5^ CH_2_CH_3_,^6^ and CH_2_C(CH_3_)_3_.^7^ Unlike the other functionalities that bind in a μ_3_-binding mode, the neopentyl groups, CH_2_C(CH_3_)_3_, in Sn_6_O_4_(OCH_2_C(CH_3_)_3_)_4_ bind to the cluster in a μ_2_-binding mode owing to steric effects.^7^

The [Sn_6_O_8_] cluster has also been observed in materials that are corrosion products on tin-alloy-containing artifacts. In Sn_6_O_4_(OH)_2_(SO_4_), a corrosion product of pewter, the [Sn_6_O_8_] clusters are joined not only by hydrogen bonding but also by covalent interactions from the bridging action of sulfate groups joining neighboring [Sn_6_O_8_] clusters. Two oxygen atoms in the sulfate moiety each bind to an octahedral face on different neighboring clusters in a μ_3_-binding mode, forming a covalently linked chain of alternating sulfate groups and [Sn_6_O_8_] clusters. The remaining two oxygen atoms in each sulfate moiety participate in hydrogen bonding with the hydroxide component of clusters in neighboring chains.^8^

Hitherto, the crystal structures of two tin(II) silicate polymorphs have been reported: a synthetic cubic polymorph, α-Sn_6_SiO_8_,^9^ and a hexagonal polymorph discovered as a corrosion product of pewter, β-Sn_6_SiO_8_.^8^ Both tin(II) silicate polymorphs exhibit framework structures in which adjacent [Sn_6_O_8_] clusters are joined by orthosilicate moieties, where the four oxygen vertices of the orthosilicate tetrahedron each bind in a μ_3_-binding mode to a different [Sn_6_O_8_] cluster. The crystal structure of α-Sn_6_SiO_8_ is analogous to zinc blende and may be described as a face-centered cubic array of [Sn_6_O_8_]^4–^ clusters with Si^4+^ cations occupying half of the tetrahedral holes.^9^ The crystal structure of β-Sn_6_SiO_8_ may be considered analogous to wurtzite.

As the tin(II) oxidation state is metastable, sufficient heating of tin(II) compounds in air results in oxidation to tin(IV). The thermal decomposition of blue-black tin(II) oxide, α-SnO, is observed between 573–773 K, ultimately forming the tin(IV) oxide cassiterite via mixed valent intermediates containing both tin(II) and tin(IV).^10,11^ Other tin(II) compounds typically thermally decompose in air at lower temperatures. The tin(II) oxyhydroxide, Sn_6_O_4_(OH)_4_, dehydrates upon heating at ca. 450 K and is ultimately oxidized to form cassiterite, proceeding via a poorly crystalline tin(II) oxide intermediate.^12^ Moreover, the thermal decomposition of tin(II) oxalate, Sn(C_2_O_4_), takes place upon heating to ca. 573 K.^13^ In contrast, the thermal decomposition of the cubic tin(II) silicate, α-Sn_6_SiO_8_, chiefly takes place over the range 873–923 K, remaining stable at significantly higher temperatures than α-SnO. It is believed that the framework structure, where [Sn_6_O_8_] clusters are joined via silicate tetrahedra, engenders the material with the observed high thermal stability and resistance to tin(II) oxidation.^9^

The synthesis and study of novel solid-state compounds containing tin(II) clusters in framework structures affords a greater understanding of these materials. Moreover, through studying their structure and thermal behavior, a better understanding of the high resistance to tin(II) oxidation may be gleaned. Herein, we report the synthesis and crystal structure of a tin(II) germanate, α-Sn_6_GeO_8_, as well as the crystal structure of a new tetragonal tin(II) silicate polymorph, γ-Sn_6_SiO_8_, formed upon cooling *α*-Sn_6_SiO_8_ below 170 K.

Although, to the extent of the authors’ knowledge, no crystal structures have been previously reported for crystalline tin(II) germanates, several studies have investigated tin(II) incorporation into germanate glass systems.^14−16^ A recent study found that tin(II) germanate glasses exhibit wide photoluminescence: a desirable property for materials in optical applications such as fiber lasers and wideband amplifiers.^14^ Bright yellow crystallites were observed in a study on a glass synthesized from SnO and GeO_2_ in a 5:3 stoichiometric ratio.^15^ Some of the reflections in the tabulated PXRD data can retrospectively be attributed to the yellow material α-Sn_6_GeO_8_, the structure of which has been elucidated in this study, although other reflections were also present in the tabulated data from at least one unknown phase.^15^ Another tin(II) germanate glass system was reported with the composition *x*SnO(1 – *x*)GeO_2_ for *x* in the range of 0.3 ≤ *x* ≤ 0.6. Neutron pair-distribution functions (NPDF) and ^119^Sn NMR spectra demonstrate that tin(II) is chiefly present in a three coordinate asymmetric environment in these glasses, where the asymmetric [SnO_3_] pyramids substitute for tetrahedral [GeO_4_] units. Increasing the tin content in the system to *x* = 0.7 and *x* = 0.8 led to the formation of a bright yellow ceramic; however, this material was not characterized, and the PXRD pattern was not published.^16^

## Experimental Section

### Synthesis of Cubic Tin(II) Germanate (α-Sn_6_GeO_8_)

The cubic tin(II) germanate, α-Sn_6_GeO_8_, was prepared by first dissolving sodium hydroxide (1.200 g, 30.0 mmol) in deionized water (30 mL). Upon dissolution of the sodium hydroxide, the following reagents were added to the solution in the order presented, once each preceding reagent had fully dissolved: oxalic acid dihydrate (0.630 g, 5.00 mmol), amorphous germanium dioxide (0.174 g, 1.67 mmol), and tin(II) chloride dihydrate (2.26 g, 10.0 mmol). The resulting solution was stirred for 30 min at ambient temperature, transferred to a Teflon-lined CEM EasyPrep vessel, and then heated in a CEM Mars 6 microwave oven at 160 °C for 30 min (not including 20 min of ramping time) at a microwave power of 600 W. The product, a bright yellow powder, was recovered by vacuum filtration, washed with deionized water, and dried overnight.

The same product may also be obtained by conventional hydrothermal synthesis, achieved by loading the homogenized mixture, once it has been stirred for 30 min, into a Teflon-lined Parr autoclave (45 mL internal volume) and heating at 160 °C in a convection oven for 10 h, rather than using microwave-assisted heating.

### Synthesis of Cubic Tin(II) Silicate (α-Sn_6_SiO_8_)

The cubic tin(II) silicate used in variable-temperature powder X-ray diffraction studies in this paper was synthesized by the method used for *α*-Sn_6_GeO_8_, as detailed above but with fumed silica (0.100 g, 1.67 mmol) in place of amorphous germanium dioxide. The product was recovered as an orange powder, in line with previous observations,^9^ by the same recovery method outlined above.

### Synthesis of Mixed Cubic and Hexagonal Tin(II) Germanate Sample (α-Sn_6_GeO_8_ and β-Sn_6_GeO_8_)

The mixed sample was prepared by first dissolving sodium hydroxide (0.200 g, 5.00 mmol) in deionized water (12 mL), followed by the addition of amorphous germanium dioxide (0.087 g, 0.83 mmol), which was stirred until fully dissolved before tin(II) oxalate was added (0.880 g, 4.26 mmol). The resulting mixture was stirred for 30 min at ambient temperature before being loaded into a Teflon-lined Parr autoclave (23 mL internal volume) and heated at 160 °C for 10 h in a convection oven. The product, a bright yellow powder, was recovered by vacuum filtration, washed with deionized water, and dried overnight.

### Materials

Fumed silica powder (0.007 μm), sodium hydroxide (≥ 98%), tin(II) chloride dihydrate (98%), and oxalic acid dihydrate (≥ 99%) were obtained from Sigma-Aldrich. Tin(II) oxalate (98%) was obtained from Alfa Aesar. Amorphous germanium dioxide was obtained from Gerald Wise & Co. Ltd. (now defunct).

### Techniques and Methods

Laboratory powder X-ray diffraction (PXRD) patterns were recorded on a Bruker D8 Advance diffractometer, in reflection geometry, equipped with a Ni-filtered Cu Kα X-ray source (λ = 1.5418 Å) and a solid-state LynxEye position sensitive detector. Scans were measured over the 2θ range 10–60° at a scan rate of 0.04° s^–1^ with a 0.02° step size.

Synchrotron PXRD patterns were collected at the Diamond Light Source on beamline I11, operating at 15 keV, using an array of 5 MAC (multiple analyzer crystal) detectors to collect data over the 2θ range 0–160°.^17^ The precise wavelength of the incident X-rays was calculated by a Pawley fit against PXRD data collected on a silicon standard.

In variable-temperature synchrotron PXRD studies, the range 100–290 K was achieved using the beamline cryostream plus sample environment. Patterns were recorded at successive 10 K increments across this range beginning at 290 K. When cooling between patterns, a 12 K min^–1^ ramp rate was employed and, once the desired temperature was achieved, a 60 s delay was performed to allow equilibration prior to commencing the data collection. During each data collection, the temperature of the sample was held at the desired temperature. Each pattern was collected for 10 min with additional longer scans performed for 30 min at both 100 and 290 K.

The temperature range 293–1034 K was studied using the beamline hot air blower sample environment. Patterns were recorded at successive 18.5 K increments across this range beginning at 293 K. When heating between patterns, a 12 K min^–1^ ramp rate was employed, and once the desired temperature was achieved, a 60 s delay was performed to allow equilibration prior to commencing the data collection. During each data collection the temperature of the sample was held at the desired temperature. Each pattern was collected for 10 min. The temperatures experienced by the sample in the hot air blower were calibrated using a platinum standard, for which lattice constants were obtained by Pawley fits over the temperature range and compared with the known thermal expansion behavior of platinum.

Rietveld refinements were performed on synchrotron PXRD patterns using GSAS-II software employing a shifted Chebyschev background model and a pseudo-Voigt profile function with a Finger–Cox–Jephcoat asymmetry correction.^18^ Prior to refinement, the PXRD data step size was rebinned from 0.001 to 0.002°. In the refinements of both α-Sn_6_GeO_8_ and γ-Sn_6_SiO_8_, the isotropic displacement parameter for the O1 environment was fixed at *U*_iso_ = 0.005 as refining this value led to unreasonably low outcomes. All images of crystal structures were produced using Vesta 3 software.^19^

The CIF files for the refined structures of α-Sn_6_GeO_8_ and γ-Sn_6_SiO_8_ have been deposited with the CCDC (Cambridge Crystallographic Data Centre) as deposition numbers 2178841 and 2178842, respectively.

Diffuse-reflectance (DR) UV–vis spectroscopy was performed on a PerkinElmer Lambda 650S spectrometer equipped with a universal reflectance accessory, which was used to measure the spectra of the solid samples. Raman spectra were recorded on a Renishaw InVia Raman microscope over the Raman shift range 100–400 cm^–1^ using a 633 nm laser excitation source. X-ray fluorescence (XRF) spectroscopy was performed with a HORIBA Jobin Yvon XGT-7000 V X-ray Analytical Microscope on the sample as a pressed pellet.

## Results and Discussion

### Synthesis of α-Sn_6_GeO_8_

**Figure 1:**
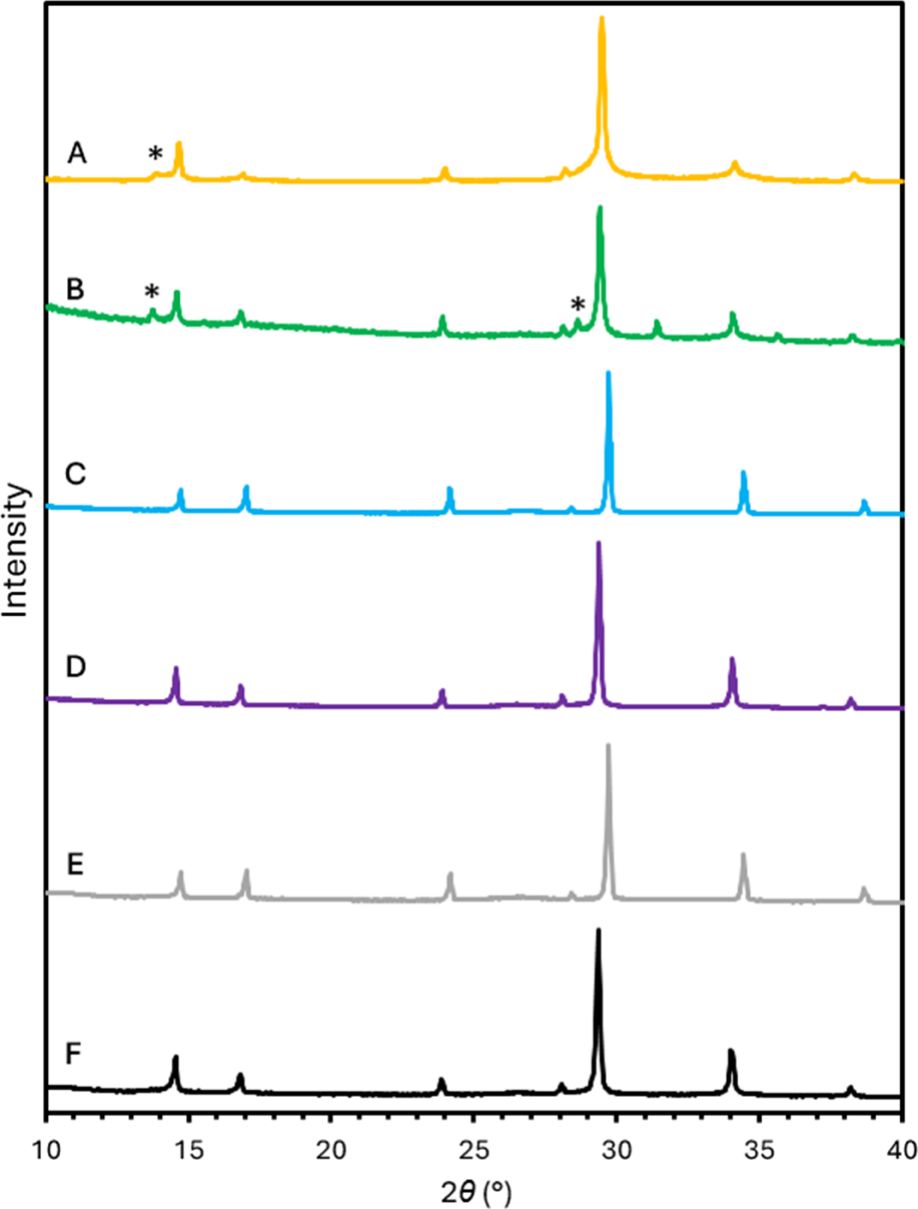
Laboratory PXRD patterns of α-Sn_6_SiO_8_ and α-Sn_6_GeO_8_-containing samples. Patterns A and B are respectively products of microwave-assisted and conventional hydrothermal syntheses on gels with the composition 10 Sn(C_2_O_4_):1.67 GeO_2_:10 NaOH:1665 H_2_O. Patterns C and E are respectively products of conventional and microwave-assisted hydrothermal syntheses on gels with the composition 10 SnCl_2_:1.67 SiO_2_:30 NaOH:5 H_2_C_2_O_4_:1695 H_2_O. Patterns D and F are respectively products of conventional and microwave-assisted hydrothermal syntheses on gels with the composition 10 SnCl_2_:1.67 GeO_2_:30 NaOH:5 H_2_C_2_O_4_:1695 H_2_O. The starred reflections correspond to β-Sn_6_GeO_8_.

Initial attempts to synthesize a tin(II) germanate proceeded by using the microwave-assisted hydrothermal method previously reported for producing α-Sn_6_SiO_8_^9^ but with a stoichiometric amount of amorphous germanium oxide (GeO_2_) in place of fumed silica (SiO_2_) such that the overall gel composition in millimoles was 10 Sn(C_2_O_4_): 1.67 GeO_2_: 10 NaOH: 1665 H_2_O. Yellow powders were obtained by this method with a PXRD pattern (pattern A in Figure 1) containing the reflections anticipated for a face-centered cubic tin(II) germanate phase, α-Sn_6_GeO_8_. The reflections observed for α-Sn_6_GeO_8_ are the same as those found in PXRD patterns of α-Sn_6_SiO_8_ but occur at lower 2θ values owing to the isomorphous substitution of germanium increasing the unit cell size. The reflections in the pattern have broad profiles: characteristic of poor crystallinity or small domain sizes within the material. An additional reflection likely originating from co-crystallization of a hexagonal tin(II) germanate polymorph, β-Sn_6_GeO_8_, was also present in the pattern at 2θ = 13.8°, corresponding to the (100) reflection.

As poor crystallinity was observed in the tin(II) germanate produced by microwave-assisted hydrothermal synthesis, conventional hydrothermal synthesis in Teflon-lined autoclaves over longer time periods was instead trialed for gels with the same composition. Heating the gel at the same temperature previously employed in the microwave, 160 °C, but for 10 h rather than 30 min in a convection oven also produced a yellow powder. The product PXRD pattern (pattern B in Figure 1) contains sharper α-Sn_6_GeO_8_ reflections intimating enhanced crystallinity, but reflections attributable to a hexagonal phase are also still observed at 2θ = 13.8 and 28.5°, corresponding to the (100) and (201) reflections, respectively.

As α-Sn_6_SiO_8_ can be synthesized as the only crystalline phase from equivalent silicate-containing gels using microwave-assisted hydrothermal synthesis,^9^ the influence of hydrothermal synthesis in a convection oven was tested on the tin(II) silicate precursor gel (10 Sn(C_2_O_4_):1.67 SiO_2_:10 NaOH:1665 H_2_O). Attempts to produce pure α-Sn_6_SiO_8_ by regular hydrothermal synthesis were unsuccessful and yielded either a mixture of hexagonal and cubic phases or instead simply recrystallized tin(II) oxalate. As it is known that pure α-Sn_6_SiO_8_ may be synthesized by microwave-assisted hydrothermal synthesis,^9^ efforts were focused on developing a regular hydrothermal method to access solely this phase with the intention of applying any successful method to the germanate system in further attempts to synthesize pure α-Sn_6_GeO_8_.

The unsuccessful attempts to synthesize only α-Sn_6_SiO_8_ by regular hydrothermal methods and the previously described syntheses of α-Sn_6_GeO_8_ containing a β-Sn_6_GeO_8_ impurity, employed tin(II) oxalate as the tin(II) source. Tin(II) oxalate is insoluble but reacts with basic solutions to form soluble species; the insolubility of tin(II) oxalate may therefore lead to inhomogeneity in reactive gels. Gels containing a soluble tin(II) source, tin(II) chloride, and varying sodium hydroxide content were therefore prepared, but in all instances, no discernible cubic or hexagonal Sn_6_SiO_8_ reflections were present in the resulting products. Further discussion on these experiments and the products obtained may be found in the Supporting Information.

As employing tin(II) oxalate led to the formation of a mixture of the Sn_6_SiO_8_ polymorphs but substituting tin(II) chloride produced different products with no trace of the Sn_6_SiO_8_ polymorphs, the oxalate ion appears to play a critical role in the formation of the Sn_6_SiO_8_ phases. An oxalate ion (p*K*_a_ = 4.27) has significantly greater basic character than a chloride ion (p*K*_a_ ≈ −7); therefore, the oxalate ion may participate as a base in the condensation reactions necessary to form the Sn_6_SiO_8_ phases. It is known from previous research and further supported in this study that discrete tin(II) clusters, Sn_6_O_4_(OH)_4_, form in solutions of tin(II) salts under alkaline conditions.^4^ Condensation reactions between aqueous silicate species and the hydroxyl components of aqueous Sn_6_O_4_(OH)_4_ clusters, however, appear to only take place in the presence of a strong base such as oxalate ions.

A gel containing both tin(II) chloride and oxalic acid, as well as an increased base content to neutralize the oxalic acid producing in situ disodium oxalate, that was heated hydrothermally at 160 °C for 10 h yielded an orange powder with α-Sn_6_SiO_8_ as the only discernible crystalline phase in the PXRD pattern (pattern C in Figure 1). Applying the same synthetic method to the germanate system yielded a bright yellow product with only reflections attributable to α-Sn_6_GeO_8_ in the PXRD pattern (pattern D in Figure 1). Heating both gels by microwave-assisted hydrothermal synthesis at 160 °C for 30 min also yields the same pure α-Sn_6_SiO_8_ and α-Sn_6_GeO_8_ products observed in the conventional hydrothermal systems, as depicted in patterns E and F, respectively, in Figure 1. The full experimental details may be found in the Experimental Section. The successful synthesis of these materials from gels containing tin(II) chloride and oxalic acid, but not from gels which did not contain oxalate ions, supports the hypothesis that oxalate plays a key role in this system in facilitating the condensation between aqueous silicate species and Sn_6_O_4_(OH)_4_ clusters.

### Characterization of α-Sn_6_GeO_8_

**Figure 2:**
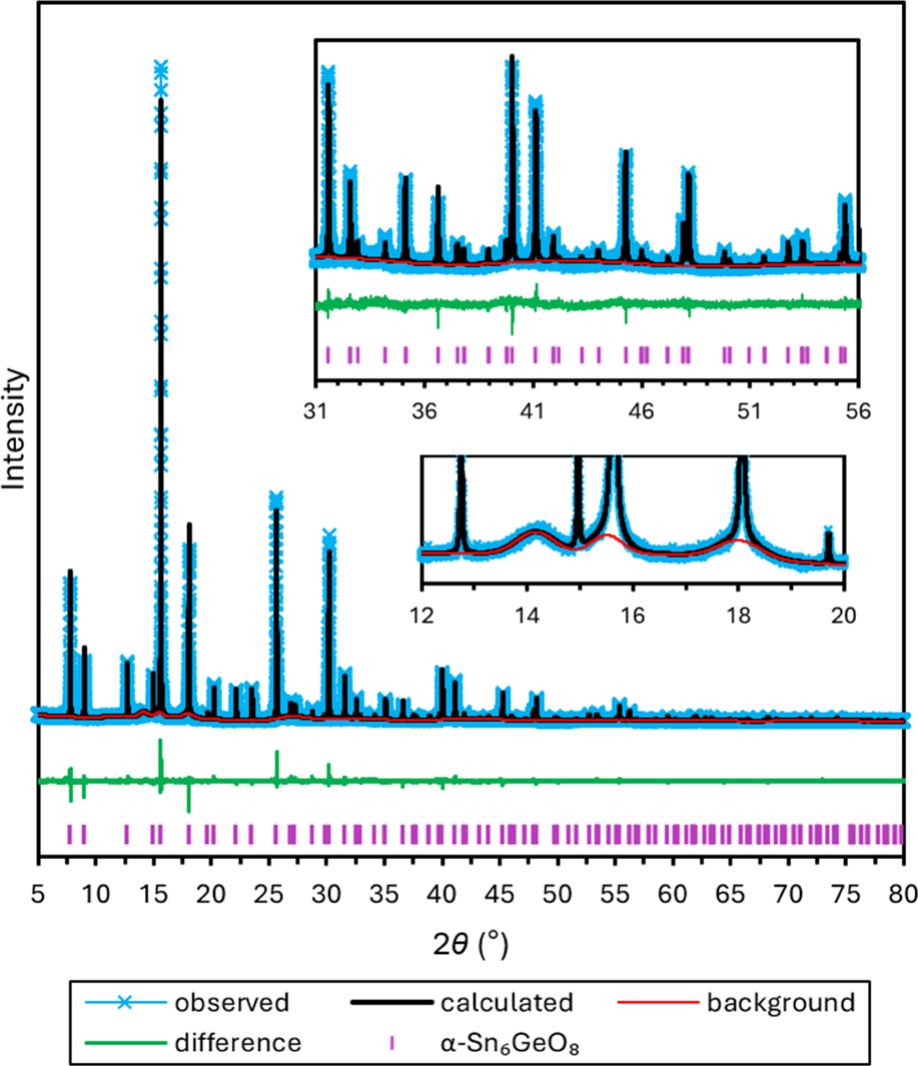
Rietveld refinement for α-Sn_6_GeO_8_ performed on synchrotron PXRD data (λ = 0.82656 Å) containing the observed pattern (light blue), calculated pattern (black), background (red), difference curve (green), and calculated peak positions (purple). The two insets show magnified regions, the top showing the magnified fit across the range of 31–56°, and the bottom showing the broad mounds modeled in the background at 2θ ≈ 14.2, 15.5, and 18.0°.

**Figure 3:**
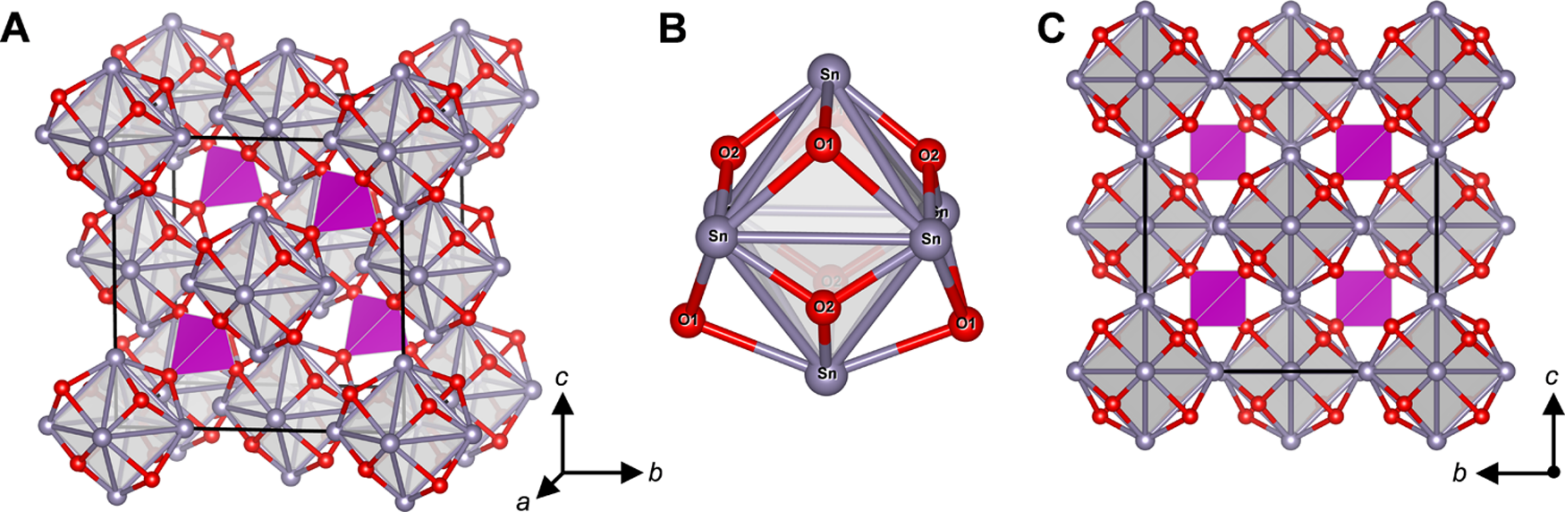
(A) Depiction of the unit cell of α-Sn_6_GeO_8_, where gray spheres correspond to Sn atoms, red spheres correspond to O atoms, the purple tetrahedra correspond to [GeO_4_] units, and the translucent gray octahedra depict the Sn_6_ arrays in the [Sn_6_O_8_] clusters. The black line demarcates the unit cell. (B) Labeled depiction of a [Sn_6_O_8_] cluster in α-Sn_6_GeO_8_. (C) Projection of the α-Sn_6_GeO_8_ structure along [100] with the same components as Figure 3A.

Following the successful synthesis of α-Sn_6_GeO_8_, its crystal structure has been solved by Rietveld refinement of PXRD data collected on beamline I11 at the Diamond Light Source synchrotron. The results of the Rietveld refinement are depicted in Figure 2 and Table 1 based on fitting 154 observed reflections over the 2θ range 5–80°. α-Sn_6_GeO_8_ is isomorphous to α-Sn_6_SiO_8_, with substitution of germanium for silicon.^9^ These structures may be described as zinc blende-type face-centered cubic arrays of [Sn_6_O_8_]^4–^ clusters with Ge^4+^ or Si^4+^ ions occupying half of the tetrahedral holes, as depicted in Figure 3. As there is only one tin environment in the crystal structure, each [Sn_6_O_8_]^4–^ cluster comprises a perfect octahedral array of tin atoms. An oxygen atom resides on each face of the Sn_6_ octahedron with two distinct environments present on alternating faces. The O2 environment is three-coordinate bonding to the three Sn atoms that form the octahedral face above which it resides in a μ_3_-binding mode, whereas O1 is a four-coordinate environment that bonds to the germanium center, forming the vertices of the [GeO_4_] tetrahedra, in addition to binding to the three tin atoms on the octahedral face in a μ_3_-binding mode. A [Sn_6_O_8_] cluster illustrating the different oxygen environments is depicted in Figure 3B. Each tin atom is four-coordinate and adopts a distorted disphenoidal geometry, binding to two O1 and two O2 atoms.

**Table 1:** Crystallographic Data for α-Sn_6_GeO_8_ and γ-Sn_6_SiO_8_

material	α-Sn_6_GeO_8_	γ-Sn_6_SiO_8_
source	synchrotron	synchrotron
chemical formula	Sn_6_GeO_8_	Sn_6_SiO_8_
formula weight (g mol^–1^)	912.882	868.218
temperature (K)	290	100
λ (Å)	0.82656	0.82656
crystal system	cubic	tetragonal
space group	*F*4̅3*m* (no. 216)	*I*4̅ (no. 82)
*a* (Å)	10.52521(2)	7.30414(6)
*c* (Å)		10.53731(6)
V (Å^3^)	1165.98(1)	562.17(1)
*Z*	4	2
χ^2^	4.10	6.57
*R*_p_^a^	0.0682	0.0724
*R*_wp_^b^	0.0888	0.0930

a .

b .

The bond lengths and angles for α-Sn_6_GeO_8_ are listed in Table 2. The Sn–O2 bond length agrees within error with the distance observed in α-Sn_6_SiO_8_; however, a small reduction in the Sn–O1 distance from 2.475(2) to 2.442(2) is observed upon replacing silicon with germanium in the structure. The reduced bond length intimates less strain in the [Sn_6_O_8_] cluster owing to the greater size of the germanium atom. The average Sn–O bond length is 2.28 Å, which is comparable to the average Sn–O bond lengths in related materials: 2.27 Å, 2.28, and 2.30 Å are the average Sn–O bond lengths in Sn_6_O_4_(OH)_4_,^4^ β-Sn_6_SiO_8_,^8^ and α-Sn_6_SiO_8_,^9^ respectively. The Sn–Sn distance from each tin atom to the nearest four tin atoms within the same cluster in α-Sn_6_GeO_8_, 3.5216(6) Å, is greater than the value observed in α-Sn_6_SiO_8_, 3.500(1) Å, further intimating less strain in the clusters of the germanate structure.

**Table 2:** Bond Lengths and Angles in α-Sn_6_GeO_8_

bond	distance (Å)	bond	angle (°)
Ge-O1	1.767(2)	O1-Sn-O1	137.8(1)
Sn-O1	2.442(2)	O1-Sn-O2	77.21(4)
Sn-O2	2.124(2)	O2-Sn-O2	104.2(1)
		O1-Ge-O1	109.47(3)

Using the utility in GSAS-II to model peaks in the background, the shifted Chebyschev background model accounts for several low broad mounds in the background at 2θ ≈ 14.2, 15.5, 18.0, and 27.1°; the first three of these may be seen in Figure 2.^18^ The broad mounds at 2θ ≈ 14.2, 18.0, and 27.1° are most likely caused by poorly crystalline cassiterite, SnO_2_, as the most intense cassiterite reflections, (110), (101), and (211), would be expected at these respective 2θ values. No cassiterite reflection would be anticipated at 2θ ≈ 15.5°, however, and the origin of this mound in the background is therefore unclear. The Sn/Ge ratio of the material has been calculated from the XRF spectrum as 6.38 (Figure S4 in the SI). As the measured Sn/Ge ratio is larger than the expected value (6.00), this supports the presence of some additional tin-containing material in α-Sn_6_GeO_8_ samples. From the excess tin in the XRF measurements, it is estimated that ca. 6% of the sample constitutes the poorly crystalline cassiterite phase.

**Figure 4:**
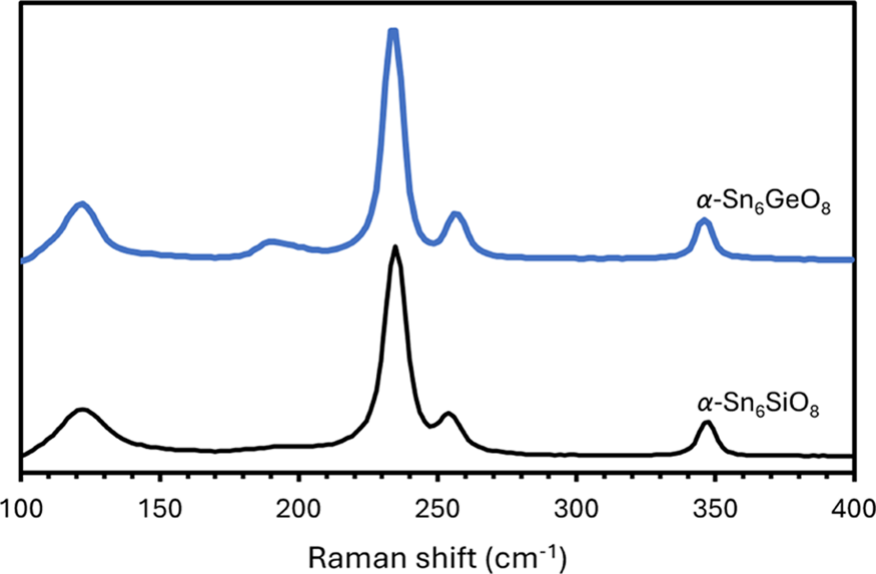
Raman spectra for α-Sn_6_GeO_8_ (blue) and α-Sn_6_SiO_8_ (black).

The Raman spectrum for α-Sn_6_GeO_8_ agrees well with the α-Sn_6_SiO_8_ spectrum (both are depicted in Figure 4) and contains peaks centered at ca. 122, 234, 256, and 346 cm^–1^, which correspond to those previously reported for α-Sn_6_SiO_8_ at the same Raman shift values within error.^9^ An additional peak of low intensity is also present at ca. 191 cm^–1^ in the α-Sn_6_GeO_8_ spectrum, which does not appear to be present in the α-Sn_6_SiO_8_ spectrum. The peaks at 122, 191, 234, and 256 cm^–1^ in the α-Sn_6_GeO_8_ spectrum also correspond to peaks observed in the Raman spectrum of Sn_6_O_4_(OH)_4_ and are therefore attributed to vibrational modes of the [Sn_6_O_8_] clusters.^20^ The absence of the peak at 348 cm^–1^ in Raman spectra of Sn_6_O_4_(OH)_4_ had led to the specious assumption that this peak was caused by the orthosilicate moieties in α-Sn_6_SiO_8_.^9^ As a corresponding peak is also observed in the α-Sn_6_GeO_8_ spectrum, it would appear more likely that this peak is caused instead by a vibrational mode of the [Sn_6_O_8_] clusters that is not observed in Sn_6_O_4_(OH)_4_, which contains discrete clusters rather than the clusters forming part of a rigid framework.

A diffuse-reflectance (DR) UV–vis spectrum has been collected on α-Sn_6_GeO_8_ (Figure S5 in the SI). A Tauc plot of the Kubelka–Munk function (Figure S6 in the SI), derived from the DR UV–vis spectrum following the method used by Patel et al.,^21^ indicates an approximate band gap of 2.65 eV for α-Sn_6_GeO_8_, in agreement with the observed yellow color of the compound. This is greater than the reported approximate band gap of α-Sn_6_SiO_8_, 2.42 eV, which is orange in color.^9^

**Figure 5:**
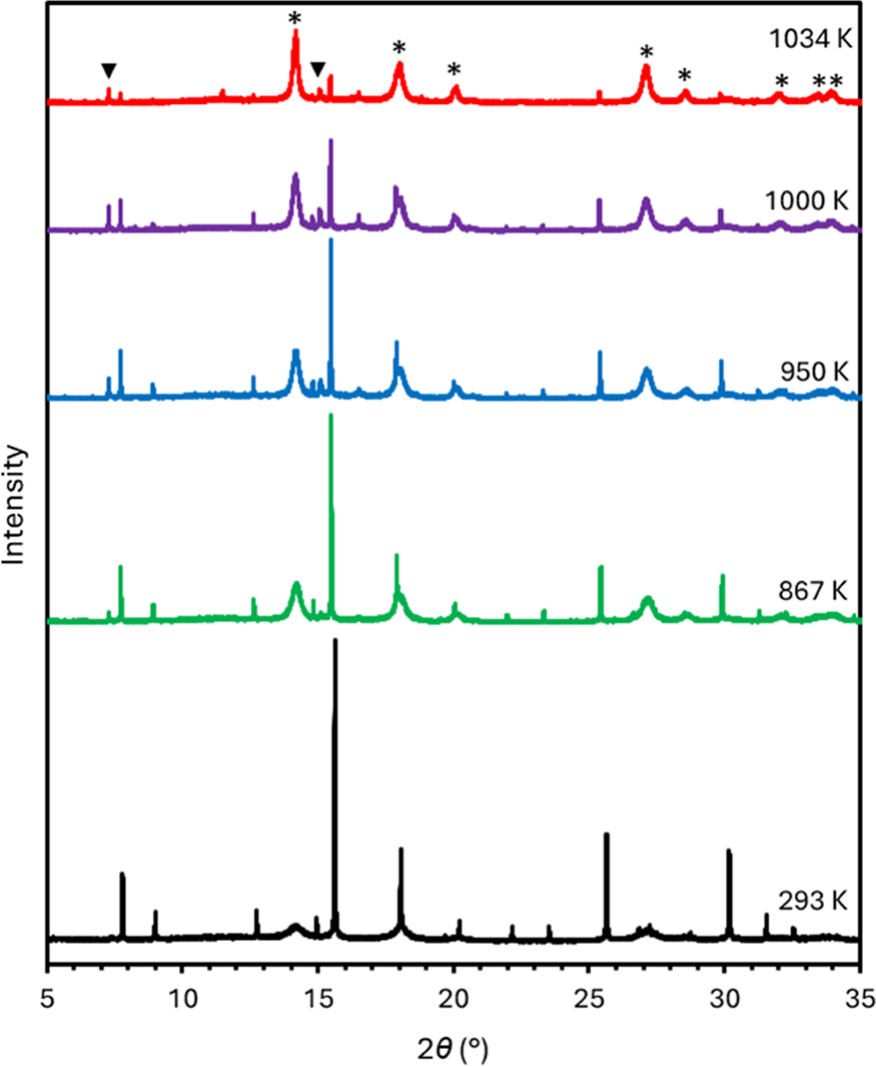
PXRD patterns at the given temperatures showing the thermal decomposition of α-Sn_6_GeO_8_ (λ = 0.82644 Å). Starred reflections correspond to cassiterite, and the reflections marked with a triangle correspond to β-Sn_6_GeO_8_.

It was previously reported that α-Sn_6_SiO_8_ is stable in air up to ca. 873 K, at which point it begins to thermally decompose with the majority of the sample oxidizing to the tin(IV) oxide cassiterite by 923 K.^9^ This thermal stability and resistance to tin(II) oxidation was noteworthy as the tin(II) oxide, α-SnO, decomposes in the range of 573–773 K.^10^ The thermal stability of α-Sn_6_GeO_8_ in air has been studied by measuring variable-temperature synchrotron PXRD patterns over the range 293–1094 K, revealing that α-Sn_6_GeO_8_ remains stable in air at 950 K, albeit with a reduction in crystallinity compared with patterns recorded at 293 K (Figure 5). Thermal decomposition is mostly observed over the range 984–1034 K, as shown in Figure 5. At 1034 K, the tin(IV) oxide, cassiterite, is the predominant phase present in the PXRD pattern; however, some α-Sn_6_GeO_8_ still remains. Hence α-Sn_6_GeO_8_ has an even greater thermal stability than the silicate analogue, in both cases ascribed to the isomorphous framework structures.

Some broad reflections attributable to cassiterite occur concomitantly with the tin(II) germanate reflections before the thermal decomposition of α-Sn_6_GeO_8_ is complete, as shown in Figure 5. The broad cassiterite reflections that are present prior to the thermal decomposition of the tin(II) germanate likely mostly originate from the crystallization of the poorly crystalline cassiterite material, which gives rise to the low intensity mounds in the background of the room temperature PXRD pattern, upon heating. Some cassiterite may also originate from the nascent thermal decomposition of α-Sn_6_GeO_8_.

Additional low intensity reflections are also present in the PXRD patterns in Figure 5 recorded at 867 K and higher temperatures that do not occur at ambient temperature and do not correspond to α-Sn_6_GeO_8_ or cassiterite reflections. The reflections at 2θ ≈ 7.3 and 15.1°, highlighted in Figure 5 with triangles, correspond respectively to the (100) and (201) reflections expected for the hexagonal tin(II) germanate polymorph, β-Sn_6_GeO_8_. The low intensity β-Sn_6_GeO_8_ reflections first appear in the PXRD pattern recorded at 851 K (Figure S7 in the SI) and continue to grow in intensity as the temperature is raised to 1000 K, but above this temperature, the intensity diminishes. The β-Sn_6_GeO_8_ produced upon heating is not considered an intermediate in the thermal decomposition of α-Sn_6_GeO_8_ as only a low concentration of the hexagonal form is produced and exists concomitantly with the more abundant cubic form. The origin of the β-Sn_6_GeO_8_ formation upon heating is not presently understood.

**Figure 6:**
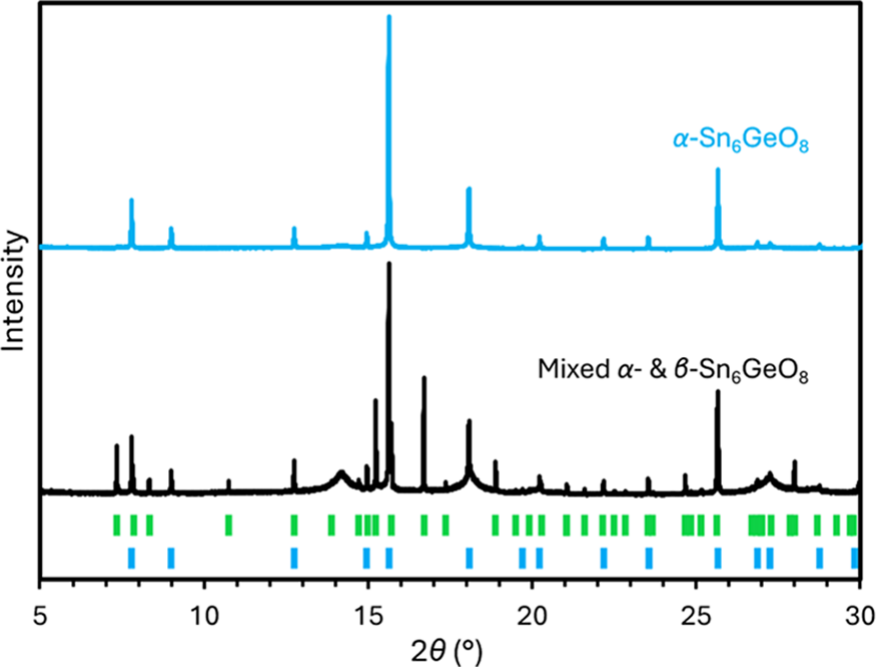
PXRD pattern of the mixed α-Sn_6_GeO_8_ and β-Sn_6_GeO_8_ sample (black) compared with a pattern of α-Sn_6_GeO_8_ (blue), with the intensities of the latter scaled down for easier comparison (λ = 0.82656 Å). Blue and green tick marks correspond to reflections anticipated for α-Sn_6_GeO_8_ and β-Sn_6_GeO_8_, respectively. The broad peaks at 2θ ≈ 14.2, 18.0, and 27.1° in the mixed-sample pattern are likely caused by poorly crystalline cassiterite.

Hydrothermal preparations that produce mixed samples of the cubic (α) and hexagonal (β) tin(II) germanate polymorphs at room temperature have been devised as described in the Experimental Section. These use tin(II) oxalate as the tin(II) source, and it was found that reducing the Sn/Ge ratio in the precursor gel from 6.00 to 5.12 led to the greatest expression of hexagonal reflections. A PXRD pattern of a mixed cubic and hexagonal sample produced by this method is presented in Figure 6.

The crystal structure of β-Sn_6_GeO_8_ has not been elucidated by Rietveld refinement due to the complex background of the mixed-sample PXRD patterns caused by the presence of significant quantities of additional poorly crystalline material, as may be observed in Figure 6. Although the structure of β-Sn_6_GeO_8_ has not been elucidated, a unit cell refinement on the hexagonal reflections using GSAS-II has revealed the lattice constants: *a* = 7.45034(5) Å and *c* = 12.08484(7) Å. It is believed that the structure is likely isomorphous with the hexagonal tin(II) silicate polymorph, β-Sn_6_SiO_8_, which adopts a wurtzite-type structure.^8^ Comparing the lattice constants for β-Sn_6_GeO_8_ with those reported for β-Sn_6_SiO_8_, *a* = 7.3742(4) Å and *c* = 11.960(1) Å,^8^ indicates that the isomorphous substitution of germanium in the structure leads to a 3.10% increase in the volume of the unit cell, which is comparable with the 3.45% increase in volume observed for α-Sn_6_GeO_8_ compared with α-Sn_6_SiO_8_.

Variable-temperature synchrotron PXRD has also been employed to study α-Sn_6_GeO_8_ and the tin(II) silicate analogue, α-Sn_6_SiO_8_, over the range of 100–290 K. Upon cooling to 100 K, α-Sn_6_GeO_8_ retains its cubic structure with a modest contraction in the lattice constant to *a* = 10.49694(2) Å, as determined by Rietveld refinement (Figure S8 in the SI). The linear thermal expansion coefficient of α-Sn_6_GeO_8_ across the temperature range 100–950 K has been calculated as α = 1.478 × 10^–5^ K^–1^ from a plot of lattice constants as a function of temperature (Figure S9 in the SI).

### Characterization of γ-Sn_6_SiO_8_

**Figure 7:**
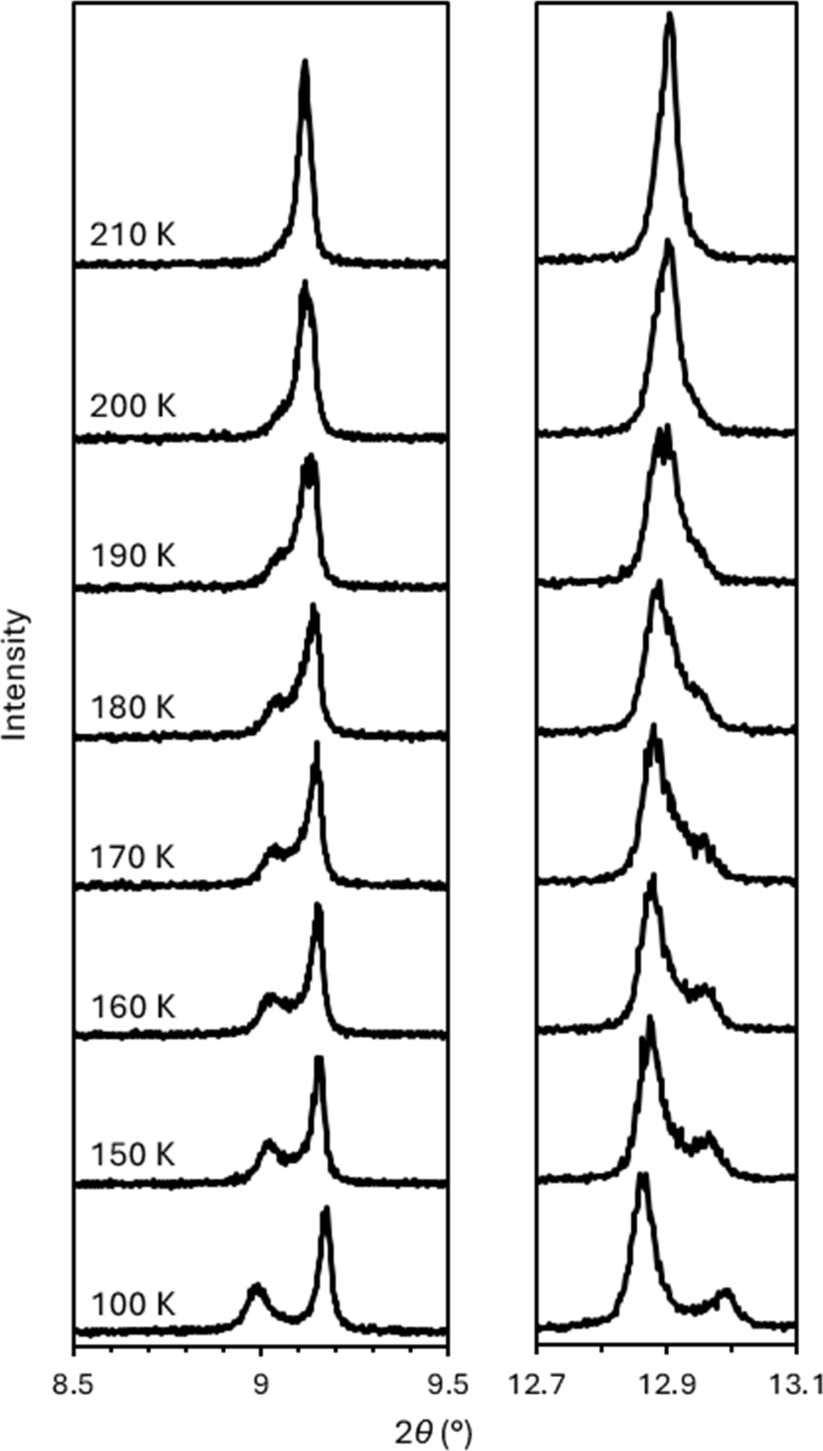
Fragments of synchrotron PXRD patterns (λ = 0.82656 Å) recorded across the temperature range 100–210 K, as labeled, showing the splitting of the (200) reflection at 2θ ≈ 9.1° (left) and the (220) reflection at 2θ ≈ 12.9° (right) as α-Sn_6_SiO_8_ transitions to γ-Sn_6_SiO_8_. The (200) reflection splits into the (002) and (110) reflections occurring at 2θ = 9.00 and 9.18°, respectively, whereas the (220) reflection splits into the (112) and (020) reflections at 2θ = 12.87 and 13.00°, respectively.

In contrast to the tin(II) germanate, when α-Sn_6_SiO_8_ is cooled, the splitting of some reflections is observed, indicating a phase transition to a tetragonal cell. The transition occurs gradually with asymmetry in the profile of some reflections first appearing at 210 K as low intensity shoulders. As the temperature is further reduced, the asymmetry becomes more pronounced. By 170 K, separate overlapping peaks are present with defined maxima, corresponding to tetragonal reflections, which continue to grow and become more distinct as the temperature is further reduced to 100 K. This series of observations is highlighted in Figure 7, which shows peak splitting in PXRD patterns recorded as the temperature is reduced from 210 to 100 K.

It is proposed that the tetragonal polymorph of Sn_6_SiO_8_ is termed γ-Sn_6_SiO_8_ to differentiate it from the cubic (α) and hexagonal (β) polymorphs. Accordingly, the tetragonal polymorph will herein be referred to as γ-Sn_6_SiO_8_.

**Figure 8:**
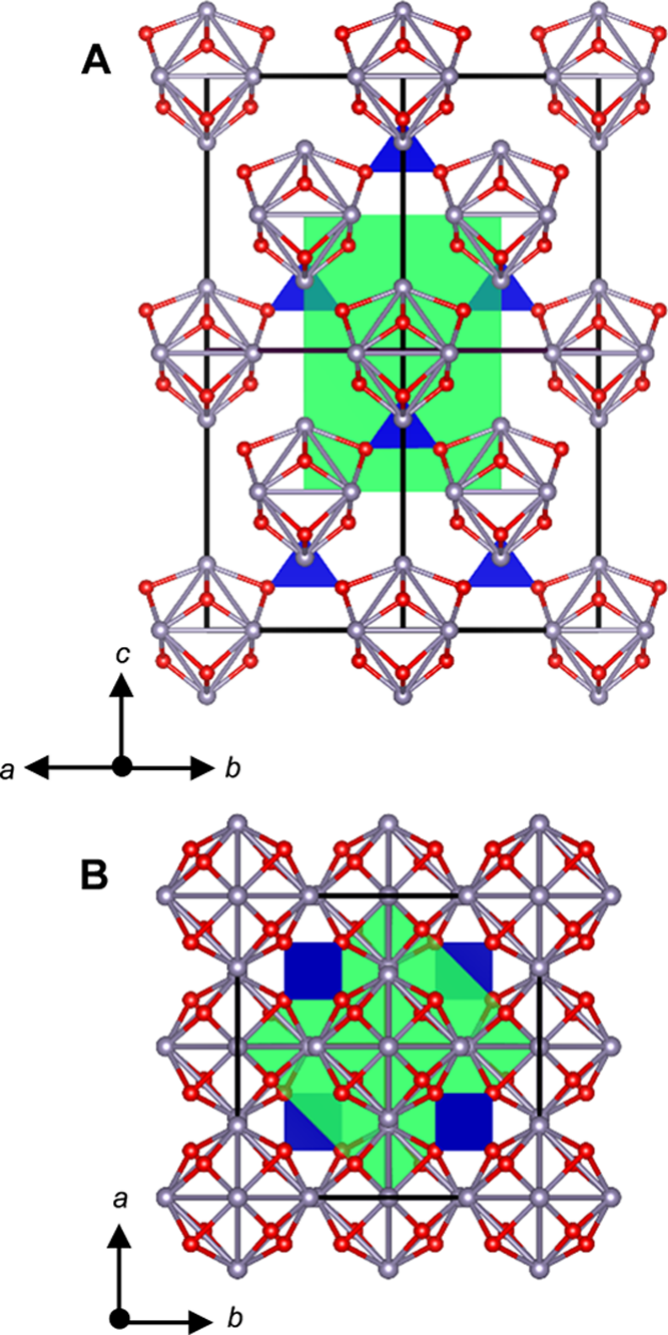
Projections of a 1 × 1 × 2 supercell of α-Sn_6_SiO_8_ where the translucent green cuboid corresponds to the tetragonal cell with its origin at (0.5, 0, 0.5) in the cubic cell. The blue tetrahedra correspond to [SiO_4_], and the red and gray spheres correspond to oxygen and tin atoms, respectively, and the black lines demarcate the cubic unit cell. (A) is a projection along [110], whereas (B) is along [001].

An indexing procedure performed in GSAS-II on the γ-Sn_6_SiO_8_ PXRD pattern, recorded at 100 K, revealed the highest figure of merit for a body-centered tetragonal cell belonging to the *I*··· extinction class. An initial model for the tetragonal phase set in the *I*4̅ space group was constructed, guided by the splitting of Wyckoff positions through maximal subgroups and the anticipated spatial relationship with the cubic α-Sn_6_SiO_8_ structure and shown in Figure 8. The volume of the tetragonal cell (562.17 Å^3^) is approximately half of the cubic cell volume (1127.17 Å^3^). The *c* length of the tetragonal cell (10.53731(6) Å) is similar to the lattice constant for the cubic cell (10.40709(2) Å); however, a Pythagorean relationship exists between the *a* parameter in the tetragonal cell (7.30414(6) Å) and the cubic lattice constant: 2(*a*_γ_^2^) ≈ *a*_α_^2^. The origin of the tetragonal cell corresponds to the position (0.5, 0, 0.5) in the cubic cell, as shown in Figure 8. Detailed discussion on the selection of the space group and construction of the model may be found in the Supporting Information.

**Figure 9:**
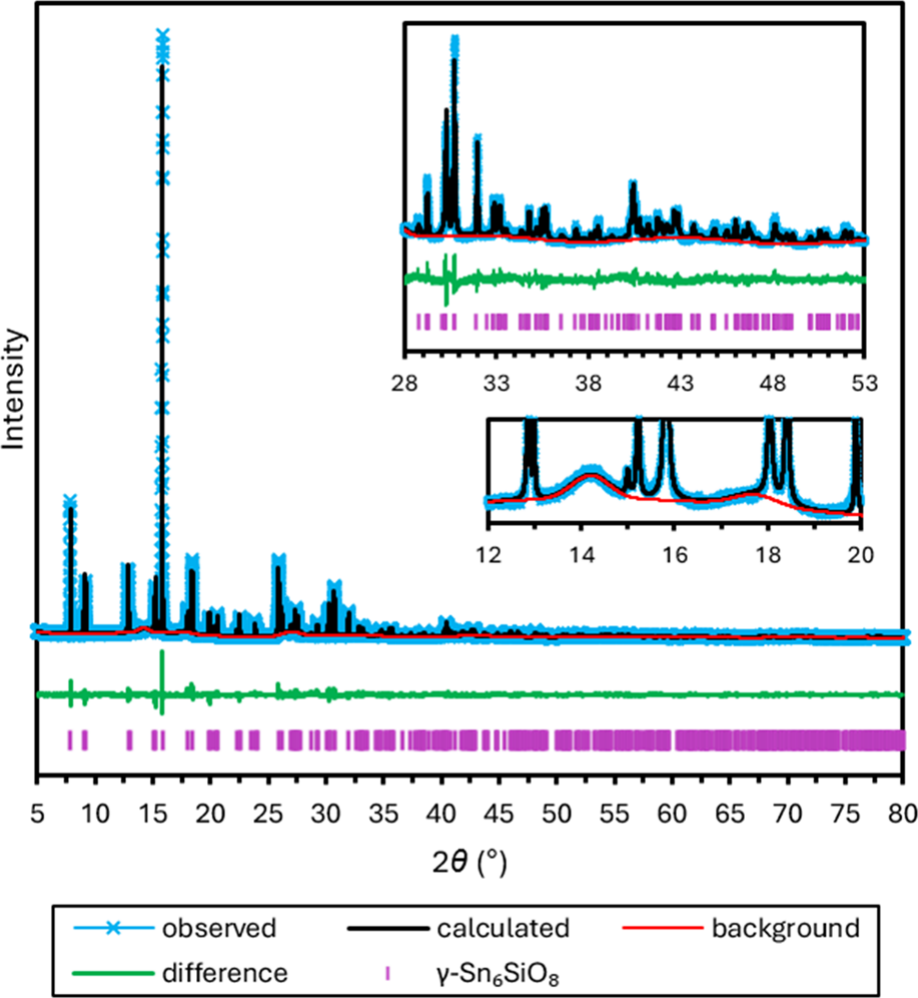
Plot of the Rietveld refinement for γ-Sn_6_SiO_8_ performed on synchrotron PXRD data (λ = 0.82656 Å) collected at 100 K, containing the observed pattern (light blue), calculated pattern (black), background (red), difference curve (green), and calculated peak positions (purple). The two insets show magnified regions, the top showing the magnified fit across the range 28–53° and the bottom showing the broad mounds modeled in the background at 2θ ≈ 14.2 and 18.0°.

Rietveld refinement of the initial model against synchrotron PXRD data collected at 100 K led to elucidation of the crystal structure of the tetragonal phase. The Rietveld refinement, depicted in Figure 9, was performed for 584 observed reflections over the 2θ range 5–80°. The applied shifted Chebyschev background model accounts for several low broad mounds in the background centered at 2θ ≈ 14.2, 17.8, and 27.1°. Corresponding mounds are also observed in the α*-*Sn_6_GeO_8_ patterns and, as discussed previously, are ascribed to poorly crystalline cassiterite, SnO_2_. The mounds at 2θ ≈ 14.2, 17.8, and 27.1° correspond respectively to the (110), (101), and (211) cassiterite reflections.

**Figure 10:**
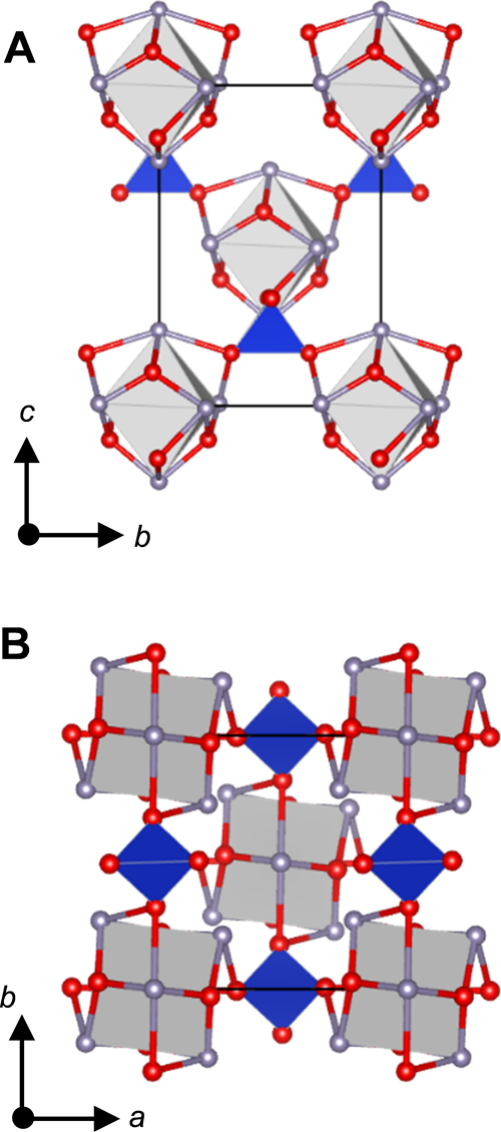
Projection of γ-Sn_6_SiO_8_ where the gray spheres correspond to Sn atoms. The gray octahedra outline the Sn_6_ octahedral array, the red atoms represent O atoms, and the blue tetrahedra represent [SiO_4_]. The black line demarcates the unit cell. (A) is a projection along [100] and (B) is along [001].

Details of the refinement are displayed in Table 1, and the refined bond lengths and angles are presented in Table 3. The γ-Sn_6_SiO_8_ structure, depicted in Figure 10, comprises a body-centered tetragonal lattice of [Sn_6_O_8_] clusters joined by orthosilicate groups. The key structural difference in γ-Sn_6_SiO_8_, when compared with α-Sn_6_SiO_8_, is the presence of two tin environments due to lower symmetry, and therefore, a range of Sn-Sn distances are present between the nearest tin atoms in the [Sn_6_O_8_] cluster spanning 3.479(2)–3.577(2) Å. Consequently, the [Sn_6_O_8_] clusters in γ-Sn_6_SiO_8_ no longer contain a perfect octahedral array of tin atoms. The Sn1 environment is located at the axial positions of the pseudo-octahedral array, whereas the Sn2 environment is located at the equatorial positions, where the defining axis is the crystallographic *c* axis. The Sn1 environment retains a four-coordinate distorted disphenoidal geometry with little variation in the Sn–O bond lengths when compared with α-Sn_6_SiO_8_. In contrast, the Sn2 environment transitions from the four-coordinate environment in α-Sn_6_SiO_8_ to a three-coordinate distorted trigonal pyramidal geometry in γ-Sn_6_SiO_8_ as one Sn2–O1 interatomic distance increases in length to 2.773(7) Å, ca. 0.27 Å longer than any previously reported Sn^2+^–O bond length. The reduced coordination is compensated by a contraction in the other Sn2–O1 bond and one of the Sn2–O2 bonds. While differences in the Sn2–O2 bond lengths are observed, the average Sn2–O2 bond length, 2.10(1) Å, is comparable with the Sn–O2 bond length in α-Sn_6_SiO_8_, 2.115(1) Å.

**Table 3:** Bond Lengths and Angles in γ-Sn_6_SiO_8_

bond	distance (Å)	bond	angle (°)
Si-O1	1.588(3)	O1-Sn1-O1	144.2(3)
Sn1-O1	2.473(6)	O1-Sn1-O2	73.1(3), 84.0(3)
Sn1-O2	2.138(7)	O2-Sn1-O2	99.9(4)
Sn2-O1	2.306(6), 2.773(7)	O1-Sn2-O1	140.9(2)
Sn2-O2	2.036(8), 2.153(8)	O1-Sn2-O2	73.2(3), 76.7(3), 78.6(3), 82.0(3)
		O2-Sn2-O2	99.7(4)
		O1-Si-O1	109.3(2), 109.8(5)

Significant anisotropic broadening is observed for the (00*l*) reflections in the γ-Sn_6_SiO_8_ PXRD pattern, which has been modeled by refining the Stephens’ microstrain broadening terms.^22^ Moreover, a restraint was applied to the Si–O1 bond set at the ideal bond length (1.625 Å) with a moderate weighting. The refined Si–O bond length, 1.588(3) Å, shows the bond contracts upon cooling from 1.648(4) Å at ambient temperature.^9^ The refined Si–O1 bond length is above the estimated lowest reasonable minimum distance for a Si–O bond: 1.560 Å.^23,24^ The contraction in the Si–O1 bond length upon cooling may be rationalized by the distortions in the [Sn_6_O_8_] clusters reducing the number of Sn^2+^ ions that coordinate to the O1 environment from three to two. The reduced coordination would lead to Si^4+^ ions exerting greater Coulombic attraction on the O1 environment, thus reducing the Si–O1 bond length.

The phase transition to γ-Sn_6_SiO_8_ is likely driven by strain incurred in the α-Sn_6_SiO_8_ structure as the material contracts with decreasing temperature, leading to unfavorable interactions that are ameliorated by the distortion of the [Sn_6_O_8_] clusters. The average intracluster Sn–Sn distance, defined as the distance from one tin atom to the nearest four tin atoms in the same cluster, and the average nearest intercluster Sn–Sn distance between neighboring clusters are presented in Table 4 for both α-Sn_6_SiO_8_ and γ-Sn_6_SiO_8_. The average intracluster distance is greater for γ-Sn_6_SiO_8_, despite an overall relative contraction in the volume of the unit cell by 0.25% compared with α-Sn_6_SiO_8_ and a reduction in the average intercluster Sn–Sn distance. The increased intracluster Sn–Sn distance indicates less strain in the clusters in γ-Sn_6_SiO_8_ and, consequently, the phase transition is likely driven by the enthalpic gain from reduced strain.

**Table 4:** Average Intracluster Sn–Sn Distance from each Tin Atom to the Nearest Four Tin Atoms within the Same Cluster and Average Intercluster Sn–Sn Distance between the Closest Tin Atoms on Neighboring Clusters for α-Sn_6_SiO_8_, γ-Sn_6_SiO_8_, and α-Sn_6_GeO_8_

	α-Sn_6_SiO_8_	γ-Sn_6_SiO_8_	α-Sn_6_GeO_8_	α-Sn_6_GeO_8_
temperature (K)	290	100	100	290
average intracluster Sn–Sn distance (Å)	3.498	3.539	3.517	3.522
average intercluster Sn–Sn distance (Å)	3.861	3.853	3.906	3.922

As a transition to a tetragonal cell is not observed upon cooling α-Sn_6_GeO_8_, this naturally implies that the crystal structure of α-Sn_6_GeO_8_ is more stable and less strained at lower temperatures than the silicon analogue α-Sn_6_SiO_8_. The enhanced stability is likely due to the greater size of the [GeO_4_] tetrahedra compared with [SiO_4_], leading to greater spacing between clusters and less strain within clusters, as highlighted by the average intracluster and intercluster Sn–Sn distances presented in Table 4 for both systems at 100 and 290 K. While a slight reduction in both the average intracluster and intercluster Sn–Sn distances is incurred upon cooling α-Sn_6_GeO_8_ from 290 to 100 K, these distances remain larger than the corresponding distances in α-Sn_6_SiO_8_ at 290 K, supporting that there is less strain in the germanate structure and rationalizing why the cubic symmetry is retained upon cooling.

## Conclusions

A method that produces only α-Sn_6_GeO_8_ as a crystalline product by microwave-assisted hydrothermal synthesis has been developed. It has also been demonstrated that this method can also employ regular heating conditions to produce the same product over longer time periods. The crystal structure of α-Sn_6_GeO_8_ has been elucidated, demonstrating that it is structurally analogous to α-Sn_6_SiO_8_ and therefore adopts a zinc blende-type structure comprising a face-centered cubic array of [Sn_6_O_8_]^4–^ clusters with Ge^4+^ occupying half of the tetrahedral holes. Variable-temperature PXRD has revealed that the tin(II) germanate has high thermal stability, enhanced even over the silicon analogue, remaining stable at 950 K and principally decomposing over the range 984–1034 K.

Variable-temperature PXRD studies on α-Sn_6_SiO_8_ have revealed a transition occurs at ca. 170 K to a tetragonal polymorph, γ-Sn_6_SiO_8_, for which the crystal structure has also been determined by Rietveld refinement, as well as the structural relationship between the two polymorphs. While γ-Sn_6_SiO_8_ forms from α-Sn_6_SiO_8_ at low temperatures, no such transition to another polymorph is observed upon cooling α-Sn_6_GeO_8_ to 100 K, indicating enhanced stability in the germanate structure.

## Supporting Information Available

Discussion on the 5.00 SnCl_2_:0.83 SiO_2_:*x* NaOH:676 H_2_O gel system, XRF spectrum of α-Sn_6_GeO_8_, DR UV–vis spectrum of α-Sn_6_GeO_8_, Tauc plot of the Kubelka–Munk function, variable-temperature PXRD patterns showing the growth of the β-Sn_6_GeO_8_ (201) reflection over the range 834–867 K, Rietveld refinement of α-Sn_6_GeO_8_ at 100 K, plot of thermal expansion behavior of α-Sn_6_GeO_8_, and discussion on the space group determination for γ-Sn_6_SiO_8_ (PDF)
