# Supplementary material for: Crystal Structures of a Cubic Tin(II) Germanate, α-Sn6GeO8, and a Tetragonal Tin(II) Silicate, γ-Sn6SiO8
Source: Inorg Chem. 2022 Sep 7;61(37):14695–704. doi: 10.1021/acs.inorgchem.2c02053 (PMC9490749; doi:10.1021/acs.inorgchem.2c02053)
Supplement: Supplementary file 1 — ic2c02053_si_001.pdf [file ic2c02053_si_001.pdf]

Supporting Information

Crystal structures of a cubic tin(II) germanate, α-Sn_6GeO_8, and a tetragonal tin(II) silicate, γ-Sn_6SiO_8

Daniel S. Parsons[a]*, Antony Nearchou[b], and Joseph A. Hriljac[a,b]

[a] Diamond Light Source, Harwell Science and Innovation Campus, Didcot, Oxon, OX11 0DE, UK.

[b] School of Chemistry, University of Birmingham, Edgbaston, Birmingham, West Midlands, B15 2TT, UK.

* daniel.parsons@diamond.ac.uk

Contents

Discussion on the 5.00 $SnCl_2$: 0.83 SiO_2: *x* NaOH: 676 H_2O gel system S-2

XRF spectrum of α-Sn_6GeO_8 S-4

Diffuse reflectance (DR) UV-Vis spectra and interpretation S-5

VT-PXRD patterns showing the formation of β-Sn_6GeO_8 S-7

Rietveld refinement of α-Sn_6GeO_8 at 100 K S-8

Thermal expansion of α-Sn_6GeO_8 S-8

Space group selection for γ-Sn_6SiO_8 S-10

Discussion on the 5.00 $SnCl_2$: 0.83 SiO_2: *x* NaOH: 676 H_2O gel system

Tin(II) chloride was substituted for tin(II) oxalate in reactive gels with varying base content, as the hydrolysis of tin(II) chloride can increase the acidity of the solution. The gels employed had the following composition (in millimoles): 5.00 $SnCl_2$: 0.83 SiO_2: *x* NaOH: 676 H_2O. Following 30 minutes of stirring, the homogenized gels were heated at 160 °C in a convection oven for 10 hours in Teflon-lined autoclaves then recovered by vacuum filtration.

The products formed from synthetic attempts employing tin(II) chloride varied depending on the base content of the gel, as shown in Table S1, which lists the product phases observed as the base content in the gel is increased. If it is assumed that the hydrolysis of one equivalent of tin(II) chloride proceeds fully to yield two equivalents of aqueous protons, correlations between the observed products and expected gel pH may be made. In gels which were expected to be acidic, i.e. an insufficient amount of sodium hydroxide was added to neutralize the acidity of the fully hydrolysed tin(II) chloride, the products were pale yellow in color and their PXRD patterns contain reflections corresponding to abhurite (Fig. S1). Abhurite is a tin(II) oxide hydroxychloride with idealized formula: $Sn_{21}O_6Cl_{16}(OH)_{14}$, that may be produced synthetically, but has also been observed as a corrosion product on the surface of tin and pewter artifacts that have spent long periods submerged in an aqueous environment.[24]

Table S1. The products formed from gels with the composition (in millimoles): 5.00 $SnCl_2$: 0.83 SiO_2: *x* NaOH: 676 H_2O. *The quoted [OH]/HCl concentration in the gel assumes full hydrolysis of tin(II) chloride takes place in solution.

x, NaOH content (mM)	[OH]/Sn	[OH]/HCl*	Product phases
5.00	1.00	0.50	Abhurite
7.50	1.50	0.75	Abhurite
10.00	2.00	1.00	Mostly α-SnO
12.50	2.50	1.25	$Sn_6O_4(OH)_4$
15.00	3.00	1.50	Poorly crystalline SnO_2

In gels with a base content equal to the concentration required to exactly neutralize all protons produced by the complete hydrolysis of tin(II) chloride ($[OH^-]$/HCl = 1.00), α-SnO is the only phase present in the product PXRD pattern (Fig. S2). Increasing the base content further to a concentration that ensures the gel would remain basic, even in the event of complete hydrolysis of tin(II) chloride ($[OH^-]$/HCl = 1.25), yields poorly crystalline tin(II) oxyhydroxide, $Sn_6O_4(OH)_4$, (Fig. S3). Further increasing the base content to $[OH^-]$/HCl = 1.50 produces a poorly crystalline material with broad peaks occurring in positions expected for the tin(IV) oxide, cassiterite, (Fig. S3).

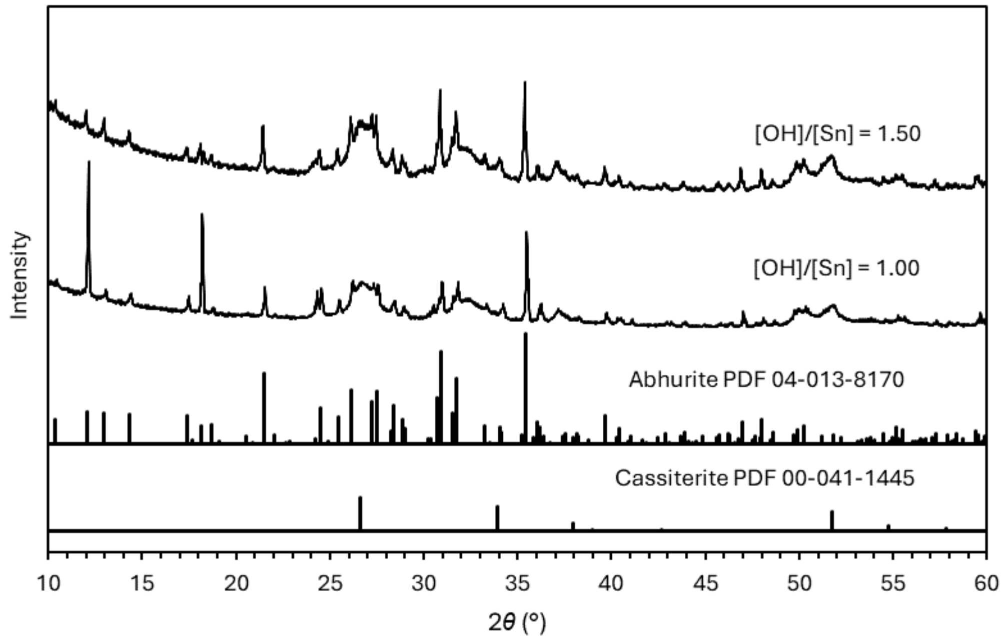

Figure S1. PXRD patterns of the products of hydrothermal synthesis in the 5.00 $SnCl_2$: 0.83 SiO_2: *x* NaOH: 676 H_2O gel system for *x* = 5.00 and *x* = 7.50.

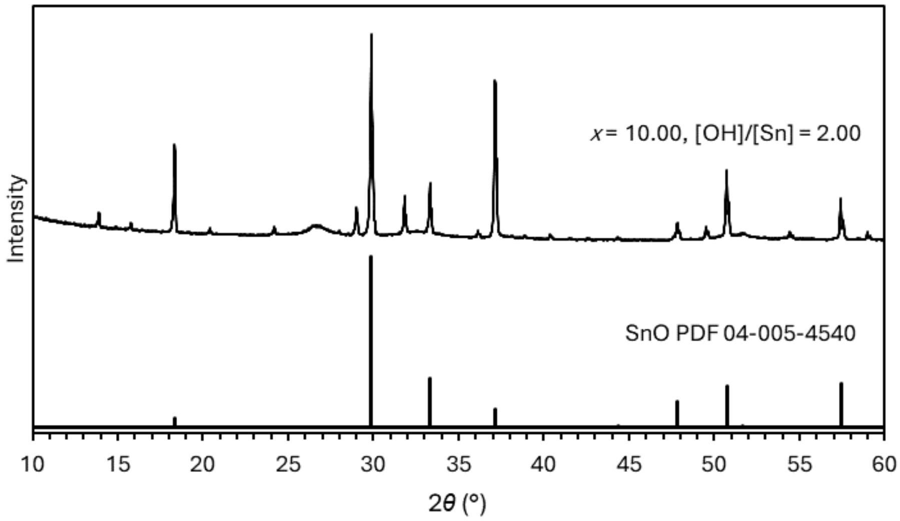

Figure S2. PXRD pattern of the product of a hydrothermal synthesis in the 5.00 $SnCl_2$: 0.83 SiO_2: *x* NaOH: 676 H_2O gel system for *x* = 10.00. Differences in relative intensity between the reference SnO pattern (PDF 04-005-4540) and the experimental PXRD pattern may be attributed to preferred orientation in the latter. Greater intensity than would be expected for 00*l* reflections is frequently encountered in PXRD patterns of SnO, owing to the platy nature of the crystallites, a consequence of the layered crystal structure.[1]

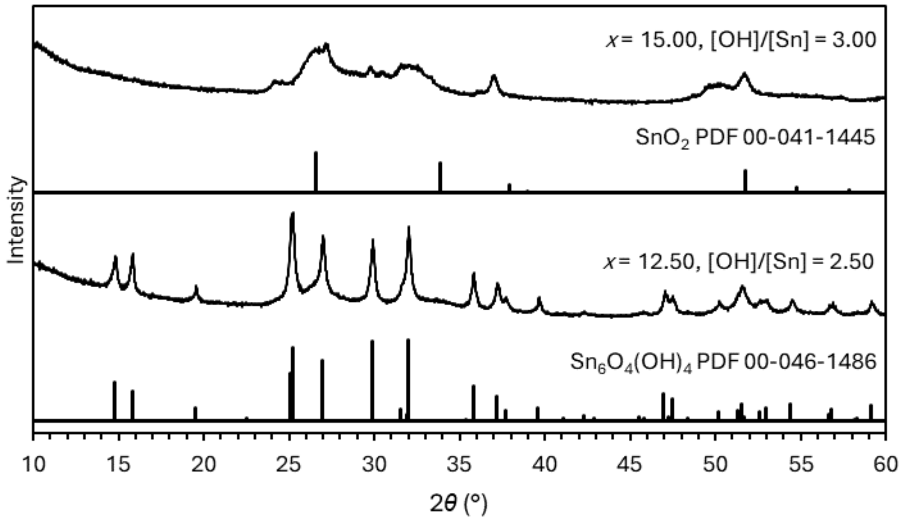

Figure S3. PXRD patterns of the products of hydrothermal synthesis in the 5.00 $SnCl_2$: 0.83 SiO_2: *x* NaOH: 676 H_2O gel system for *x* = 12.50 and *x* = 15.00.

XRF spectrum of α-Sn_6GeO_8

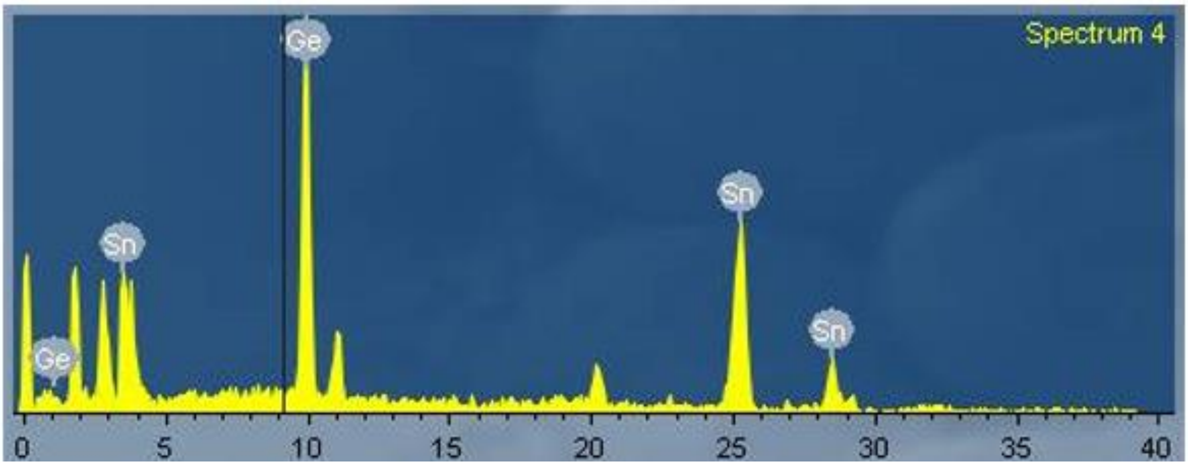

Figure S4. XRF spectrum of α-Sn_6GeO_8 recorded on a HORIBA Jobin Yvon XGT-7000V X-Ray Analytical Microscope. The energies on the *x*-axis are in keV. The Ge and Sn peaks are labelled in the figure. The unlabelled peaks in the spectrum correspond to peaks for Rh, from the X-ray source in the instrument, and Si, from the glass slide on which the sample pellet was mounted.

Diffuse reflectance (DR) UV-Vis spectra and interpretation

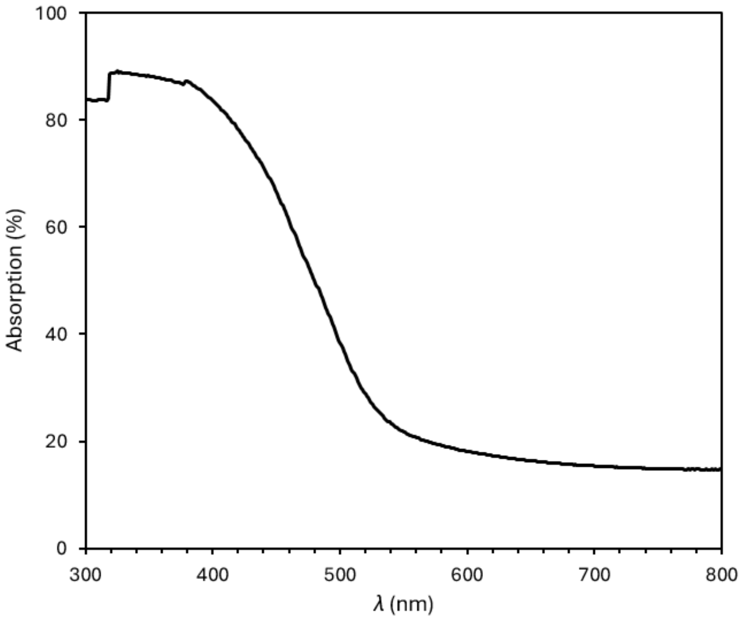

Figure S5. Diffuse reflectance UV-Vis spectrum of α-Sn_6GeO_8. The discontinuity at *ca*. 320 nm is an instrumental artifact owing to the changeover from the deuterium source to the tungsten source in the instrument at this wavelength.

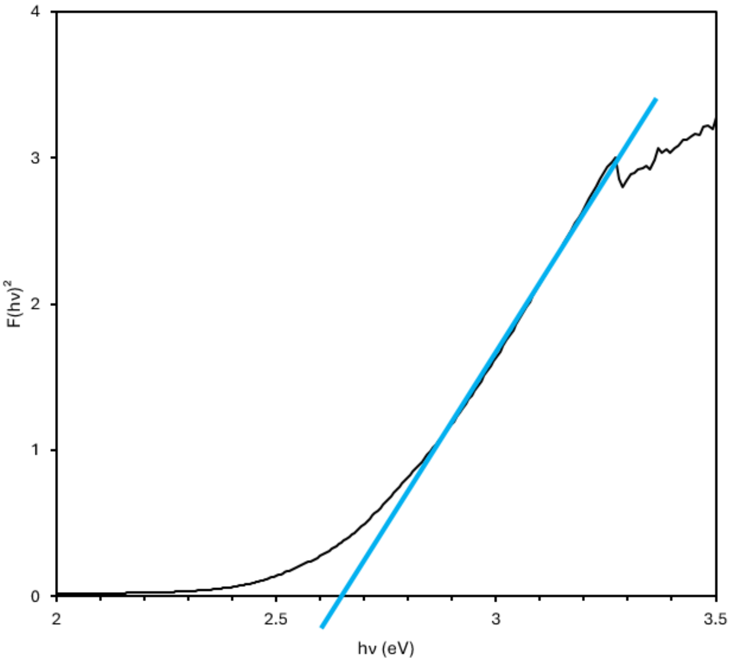

Figure S6. A Tauc plot of the Kubelka-Munk function.

The band gap (2.65 eV) was estimated from the Tauc plot of the Kubelka-Munk function (Figure S7) following the method reported by Patel *et al.*[21]. The Kubelka-Munk function was calculated by

Equation S1 where F(hv) and R(hv) are the Kubelka-Munk function and the reflectance, respectively, at given energy hv. The Tauc plot is obtained by plotting $F(hv)^2$ as a function of hv.

$$\text{Eq. S1.} \quad F(h\nu) = \frac{(1-R(h\nu))^2}{2R(h\nu)}$$

VT-PXRD patterns showing the formation of *β*-Sn_6GeO_8

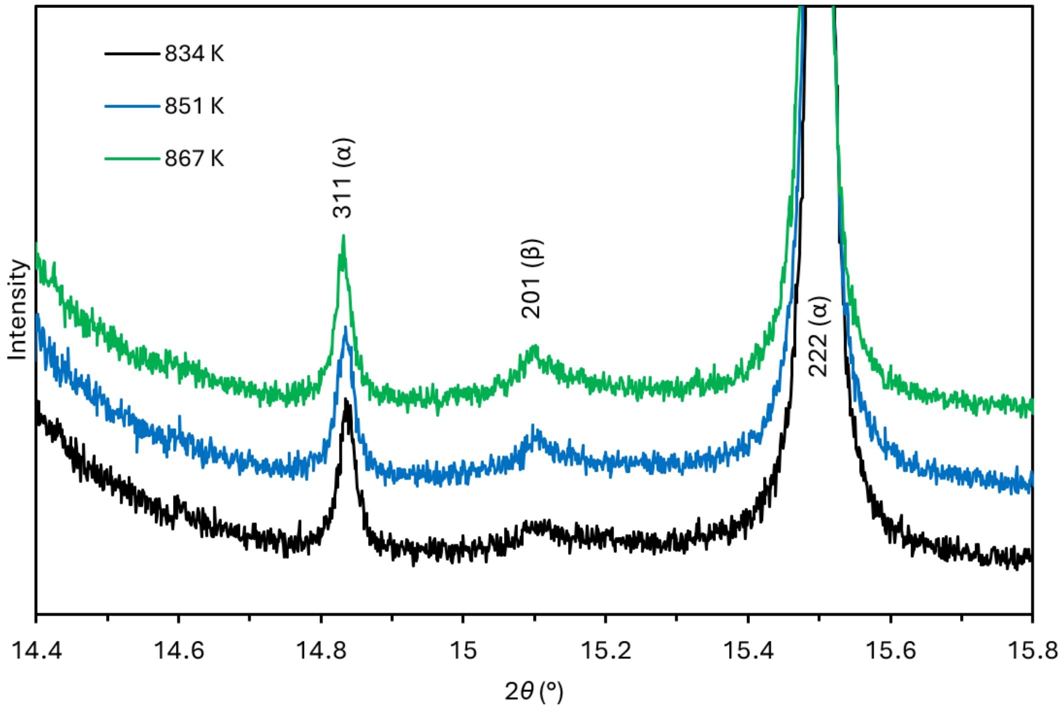

Figure S7. Variable temperature synchrotron PXRD patterns showing the formation of the (201) reflection ($2\vartheta \approx 15.1°$) of *β*-Sn_6GeO_8 over the temperature range 834 – 867 K. The (311) and (222) reflections of *α*-Sn_6GeO_8 are also shown at $2\vartheta \approx 14.85°$ and $2\vartheta \approx 15.5°$, respectively.

Rietveld refinement of α-Sn_6GeO_8 at 100 K

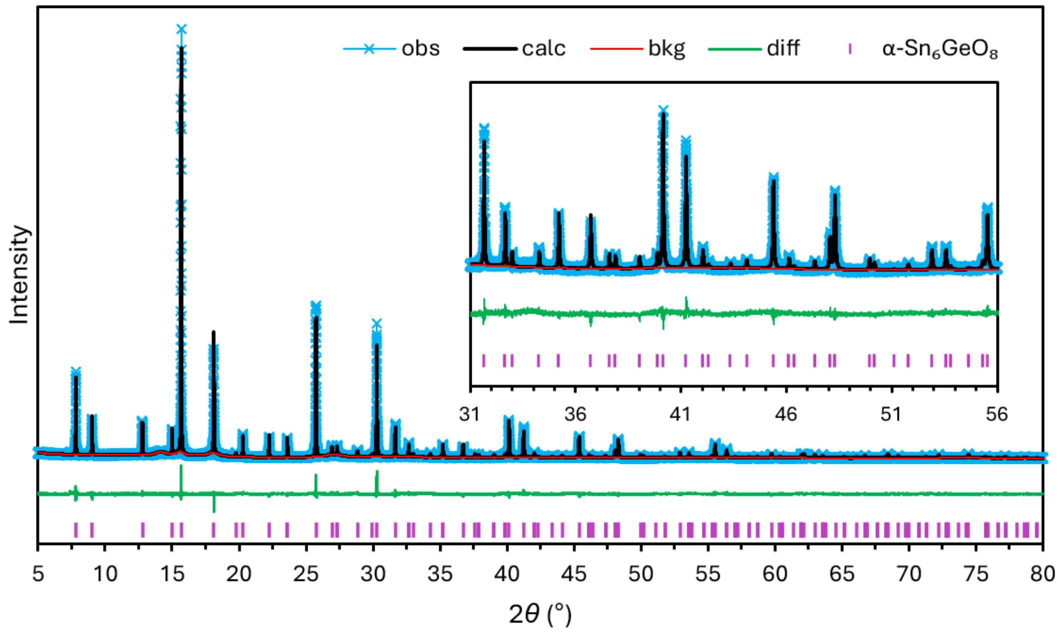

Figure S8. Rietveld refinement of α-Sn_6GeO_8 at 100 K (R_p = 0.0703, R_{wp} = 0.0931).

Thermal expansion of α-Sn_6GeO_8

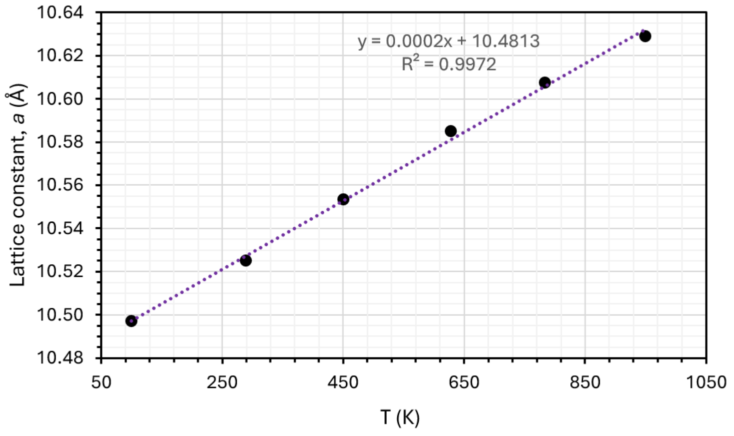

Figure S9. A plot of lattice constant as a function of temperature for α-Sn_6GeO_8. The equation for the line of best fit and the R^2 value for the fit are included on the plot. The lattice constants at 100 K and 290 K were determined by Rietveld refinements of 30-minute synchrotron PXRD data collections. The lattice constants at 451 K, 628 K, 784 K and 950 K were determined by Pawley fits, performed in GSAS-II, for 10-minute synchrotron PXRD data collections.

Space group selection for γ-Sn_6SiO_8

An indexing procedure performed in GSAS-II on a γ-Sn_6SiO_8 PXRD pattern, recorded at 100 K, revealed the highest figure of merit for a body-centred tetragonal cell belonging to the *I*--- extinction class. There are 8 space groups in the *I*--- extinction class, which by definition all exhibit the same systematic absences. These eight space groups are *I*4 (79), $I\bar{4}$ (82), *I*4/*m* (87), *I*422 (97), *I*4*mm* (107), $I\bar{4}2m$ (121), $I\bar{4}m2$ (119) and *I*4/*mmm* (139), all of which contain either a 4-rotation axis or a $\bar{4}$ inversion axis. In α-Sn_6SiO_8, a $\bar{4}$ inversion axis is present but there is no 4-rotation axis, due to the inequivalent substituents on the octahedral faces of the [Sn_6O_8] clusters, preventing this symmetry element from existing in the structure. As inequivalent oxygen environments within the [Sn_6O_8] clusters would be expected in any framework structure that connects these clusters via cationic centres, the space groups containing a 4-rotation axis may be dismissed, leaving 3 possible space groups: $I\bar{4}$ (82), $I\bar{4}2m$ (119) and $I\bar{4}m2$ (121).

The critical difference between the 3 candidate space groups, $I\bar{4}$ (82), $I\bar{4}2m$ (119) and $I\bar{4}m2$ (121), is the presence of a mirror plane and rotation axis in the latter two, whereas these symmetry elements are absent in $I\bar{4}$. Mirror planes are present in α-Sn_6SiO_8, which occur co-incident with the {110} lattice planes. 3-rotation axes are also present in α-Sn_6SiO_8, which run in the ⟨111⟩ directions, such that each rotation axis in the structure runs parallel to a mirror plane, as highlighted in Figure S12. The highest order rotation axis in the space groups $I\bar{4}2m$ and $I\bar{4}m2$ is a 2-rotation axis, therefore the 3-rotation axis is not preserved in the phase transition from the cubic cell to the tetragonal. Any structural change which eliminates the 3-rotation axis is also likely to eliminate the mirror planes which run parallel to them; therefore $I\bar{4}$ appeared the most plausible space group for γ-Sn_6SiO_8 and was chosen as the space group with which to construct an initial model to be used in a Rietveld refinement.

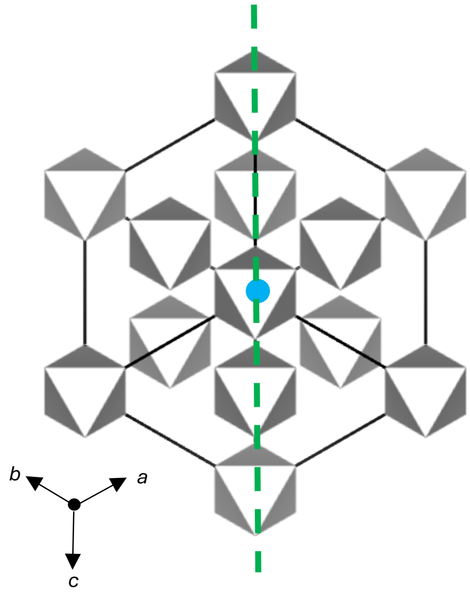

Figure S10. A depiction of α-Sn_6SiO_8 along [111] showing only the grey octahedra that trace the Sn_6 octahedral arrays. The green dashed line represents one of the mirror planes in the structure – in this case coincident with the (-101) plane. The blue circle shows the 3-rotation axis about the central octahedron that run parallel with the mirror plane.

Construction of the model was aided by using maximal subgroup-minimal supergroup relationships, as $F\bar{4}3m$ is a minimal supergroup of $I\bar{4}m2$, which is in turn a minimal supergroup of $I\bar{4}$. Accordingly, the splitting of Wyckoff positions through maximal subgroups guided construction of the tetragonal

model, in addition to considerations of the likely spatial relationship between the tetragonal cell and the cubic predecessor.

A model may also be constructed in the $I\bar{4}m2$ space group using the maximal subgroup relationship with $F\bar{4}3m$; however, refining such a model against the experimental pattern leads to poor fits (R_{wp} = 16.35 %). Moreover, a refined model set in $I\bar{4}m2$ provides no calculated intensity for several observed reflections, including the (211) and (411) reflections. Ultimately, this further supports that $I\bar{4}$ is the true space group. The critical difference between models constructed in $I\bar{4}m2$ and $I\bar{4}$ are the site symmetries in the former and the way this impacts the structure. In models set in the $I\bar{4}$ space group, the Sn2, O1 and O2 sites occupy the 8*g* general position, but in an $I\bar{4}m2$ model, these atoms must reside on special positions: with Sn2 residing on the 8*g* position (with ..2 point symmetry) and O1 and O2 both residing on the 8*i* position (.m. point symmetry). Accordingly, the point symmetries of the special positions impose spatial limitations on where these atoms may reside in the refined $I\bar{4}m2$ model. For the Sn2 site which requires atoms to be located on (*x*,*x*,0) positions, all four Sn2 atoms in each cluster must reside within the same plane at a constant *z* value which prevents the distortions in the cluster that are observed in the refined $I\bar{4}$ model (R_{wp} = 9.30 %) from occurring in the $I\bar{4}m2$ model (R_{wp} = 16.35 %).
